# Humor interventions in psychotherapy and their effect on levels of depression and anxiety in adult clients, a systematic review

**DOI:** 10.3389/fpsyt.2022.1049476

**Published:** 2023-01-04

**Authors:** Federico S. M. Sarink, José M. García-Montes

**Affiliations:** Department of Health, Psychology and Psychiatry, University of Almería, Almería, Spain

**Keywords:** humor, psychotherapy, depression, anxiety, positive psychology, confusion, alternative framework

## Abstract

**Introduction:**

Humor as a valuable construct in psychology has been the subject of much discussion for many years and has received increased attention more recently in the field of positive psychology. However, empirical research on the application of humor in a clinical setting with depressed or anxious clients has been difficult to discover. Because of the potential benefits and the low costs of providing humorous interventions, our goal was to give an overview of the studies conducted in psychotherapy and to show the effect of humor on the levels of depression and anxiety symptoms. Furthermore, we wanted to assess the empiric support of humor as a clinical intervention in psychotherapy according to the SIGN system.

**Methods:**

We used the PRISMA guidelines. Because of the differences in the design of the 10 included studies, it was not possible to perform a meta-analysis.

**Results:**

Results from studies performed in seven different countries show that humorous interventions can have significant positive effects on symptoms of depression and anxiety. The results also confirm the prior observation that empirical research in the field is based on different designs with different populations and different methods of translating the abstract concept of humor into measurable observations. The results need to be considered with caution because of the methodological limitations of the research to date.

**Discussion:**

Some authors advocate for an integrative approach to continue research on humor in psychotherapy. It is our recommendation to first focus on the separate aspects of humor and to conduct research based on sound methodology. To initiate wider research to the application of humor in psychotherapy, we propose an approach to humorous interventions based on surprise and confusion which can help clients to search for an alternative framework to resolve the confusion and therefore promote taking on new perspectives and distancing themselves from the actual problem.

## Introduction

Theorizing about humor can be traced back as far as Aristotle, according to Martin (1). However, Martin found that a common observation made by most authors in the field of humor is that although it could be important in the different disciplines of human sciences, psychology has put little importance on this subject up to now. Gremigni (2) states that literature about the role of humor has been focused more on its use as a coping strategy than on its use as a tool for therapists. Martin (1) adds that a sense of humor as a concept has grown in importance over the years. Siurana Aparisi (3) defines the concept of humor as the capacity to perceive or present something as comical and as a consequence activate the emotion of hilarity. When directing the focus on “a sense of humor” Falkenberg et al. (4) found that a definition could be borrowed from personality psychology in which humor is a personality characteristic that contains different components. These correspond with the components that Martin mentions (1, 2) in his multidimensional conceptualization, which comprises a cognitive ability, an aesthetic response, a habitual behavior pattern, an emotion-related temperament trait, an attitude, and a coping strategy or defense mechanism.

Although many of the writings on humor in psychotherapy have been dedicated to a sense of humor, there have been therapists who have written about strategies to consciously incorporate humor as a therapeutic tool (5–13). O’Brien (10) for example used the acronym SLAP (Surprise, Light-heartedness, Absurdity, and Perspective development) to instruct therapists in his study to deliberately take a humorous stance in the therapy sessions. Studying the possible benefits of positive psychology interventions on the wellbeing of participants, Wellenzohn et al. (5, 12, 13) and Crawford and Caltabiano (11) tested several humorous interventions and found results confirming happiness enhancing and depressive feelings decreasing effects. From another perspective than the positive psychology, Ellis (14) has written how he uses humor in his Rational Emotive Therapy (RET) in different ways with the goal of challenging the “crazy ideas” of his clients directly. The rationale behind this, according to Ellis, is that human disturbance is mainly based on “the exaggeration of the importance of the seriousness of things.” One of the main methods of helping the client could be the “ripping up of the exaggerations by humorous counter-exaggerations of the therapist.” Sarink (7) points out that humor is an essential ingredient when using the paradoxical techniques as applied in provocative therapy and in an approach like the logotherapy of Frankl (15). McGhee took a different approach and designed a method to teach clients to be more humorous and therefore be able to take on a humorous perspective on the problems they are dealing with (11, 16, 17). Even though the above authors use humor as a therapeutical technique, the problem remains that there is a scarcity of empirical research. Without more empirical support, the application of humor as a therapeutic intervention might be limited to merely an interesting topic to discuss. For clinicians to learn about the possibilities of humor interventions, more convincing data is required instead of theoretical assumptions and anecdotal evidence.

To be able to investigate humor in the clinical field, a starting point is to find a definition of what humor in therapy exactly is. This appears to be a challenging task because of the different perspectives researchers have (2, 10). Authors have tried to explain too many different types of humor, while it still is questionable if a comprehensive theory of humor is possible at all (1). Although empirical research to humor production is scanty, when looking for a definition of “humorous interventions in psychotherapy” authors have proposed several aspects. Martin (1) for example considers the humor process as divided into four essential components: a social context; a cognitive-perceptual process; an emotional response; and the vocal-behavioral expression of laughter.

In the last decades, the perspective on humor in psychotherapy has mainly been one that explores the beneficial aspects. Sarink (7) describes how in earlier days some authors were advocating against the use of humor in the clinical setting (18). One of the problems of the use of humor by the therapist might be the blocking of the client’s flow of feeling and thinking. Another risk might be the disguised hostility of the therapist using humor. Moreover, humor could lead the client to doubt whether to take the therapist seriously or not.

Other authors are not negative about the use of humor but propose that therapists should always use the frame of reference of the clients, and the humor should not be directed at the clients as persons, but at their non-functional ideas (7, 14). Panichelli (19) suggests that if therapists transmit their esteem and respect for the clients and their suffering, the use of humor is a situation of joining. Joining is seen as a fundamental aspect of establishing a therapeutic relationship which is necessary for therapeutic change.

### The present study

Based on the current literature, we have found that the application of humor in psychotherapy is mainly considered to be useful. But with so many different viewpoints on humor and its application in mental health care, it is important to evaluate the empirical evidence on the subject. This review intends to give an overview of the studies on the application of humor as a therapeutic intervention that have been conducted in psychotherapy applied to clients with depressive or anxiety symptoms. Humor as a therapeutical intervention in psychotherapy has not been the subject of a systemic review of the literature so far.

Our second goal is to show the effect of humor on the levels of depression and anxiety symptoms, not so much whether they are effective in decreasing the symptoms.

Furthermore, we want to assess the empiric support of humor as a clinical intervention in psychotherapy according to the SIGN system (20).

Some systematic reviews focused on humor-related constructs like banter (6) and laughter (21, 22). Although banter and laughter share conceptual similarities with humor, it is important to note that although laughter has been used frequently as a measure of (perceived) humor, laughter is not the same as humor. Sometimes we do not laugh although we find something funny. At other times, we laugh, not because of humor but because we feel guilt, anxiety, or nervousness (23). The same can be said about banter. Brooks et al. (6) found that when they split down the concept of banter in its main components, overlap with humor was found in the literature. Therefore, in this systematic review, we consider all humorous interventions and not just interventions based on banter or laughter.

## Materials and methods

### Design

A systematic literature review of qualitative and quantitative research was performed in September and October of 2021. New publications were checked until June of 2022. We used the PRISMA guideline (24).

### Review protocol

A review protocol was written before starting the literature search and was evaluated and approved by the director of the Ph.D thesis. Because this was not a clinical study, we did not preregister. Modifications and reports of the research process were added in a modified version of the review protocol. Both are accessible through: http://humorinpsychotherapy.com/review-protocol/.

### Eligibility criteria

Based on an early-stage orientation on the subject, our initial impression was that it would be difficult to find enough studies that would be eligible if we would only select for the variables: humor interventions and the effect on psychological flexibility, depression, and anxiety.^1^ This was confirmed when we asked S. Hayes to provide scientific literature on these criteria. Hayes is a specialist in psychological flexibility through his work on the Acceptance and Commitment Therapy (ACT). He could provide us with only three articles, which did not meet the eligibility criteria for this systematic review. Therefore, to prevent losing information that could be relevant to our research we decided to look for humor in the broadest sense of the word.

We used the following inclusion and exclusion criteria:

### Inclusion

(a) No restrictions were imposed on the date of publication, (b) published, as well as unpublished, investigations were included, (c) articles in the English, Spanish, and Dutch language were included, (d) participants were adults (18–65 years). Regarding the type of studies we included (e) meta-analysis and systematic reviews, (f) randomized controlled trials, (g) observational studies with the principal focus on humor as a therapeutic intervention, (h) case studies with the principal focus on humor as a therapeutic intervention, (i) cross-sectional studies which contain humor as at least a variable of the personality of the therapist or client, (j) correlational studies that relate humor with one or more variables relevant to therapeutical interventions. Regarding the type of interventions, we included studies with (k) the focus on applying any type of humor in therapy, on the influence of humor on the psychopathology of the clients and/or their personality, (l) with interventions in an individual and/or group setting, and (m) the length of the interventions investigated should be at least a minimum of three sessions (brief therapy). There was no maximum to the number of sessions. Finally, regarding the type of outcome measures, we searched for studies that investigated (n) the role of humor in increasing the psychological flexibility of clients and the effect on their depression or anxiety. And (o) the outcome measures should have been derived from standardized and validated scales.

### Exclusion

(p) Studies that focused on participants who are cognitively impaired due to, for example, autism, dementia, or an accident which led to brain damage, (q) studies that focused on participants from the main public, without being diagnosed with a depression and/or an anxiety disorder, and (r) studies performed outside a therapeutical setting.

### Study selection

#### Information sources

To search for relevant studies, we used the databases of the library of the University of Almería. We selected SCOPUS and Proquest as the main databases to start our search. These databases contain among others the following databases: Psychinfo, Medline/Pubmed, Psycarticles, PsycBOOKS, PsycTESTS, and Psychology database. Furthermore, we used the database Psicodoc because it contains studies in the Spanish language, and the database Narcis because it contains studies in the Dutch language.

When the full text article was not directly available after the initial search, we utilized the library of the University of Almería. The website ResearchGate also proved useful to request the full text articles from the authors themselves. On other occasions, we contacted the authors directly or asked for suggestions on more studies on the topic.

The search strategy that we used for all the databases can also be found in the online aforementioned review protocol.

#### Final study selection

Because of the scarcity of research we found that was consistent in method and target group, case studies were excluded because they would only provide a blurred vision on the subject, adding more anecdotal evidence/theorizing instead of a methodologically sound and clear contribution. Other studies were rejected because (a) they had nothing to do with humor, but for example with humoral body fluids or with humor in the sense of “mood,” (b) because they were addressing a setting in the (somatic) health psychology, or (c) they addressed research conducted in a coaching setting which both differ from the clinical psychology. (d) Many studies included humor as “a sense of humor” as part of coping without “humor” being the main focus of the research.

The entire process of searching for studies through selecting based on the eligibility criteria was performed by the main author and monitored by the second author.

**FIGURE 1:**
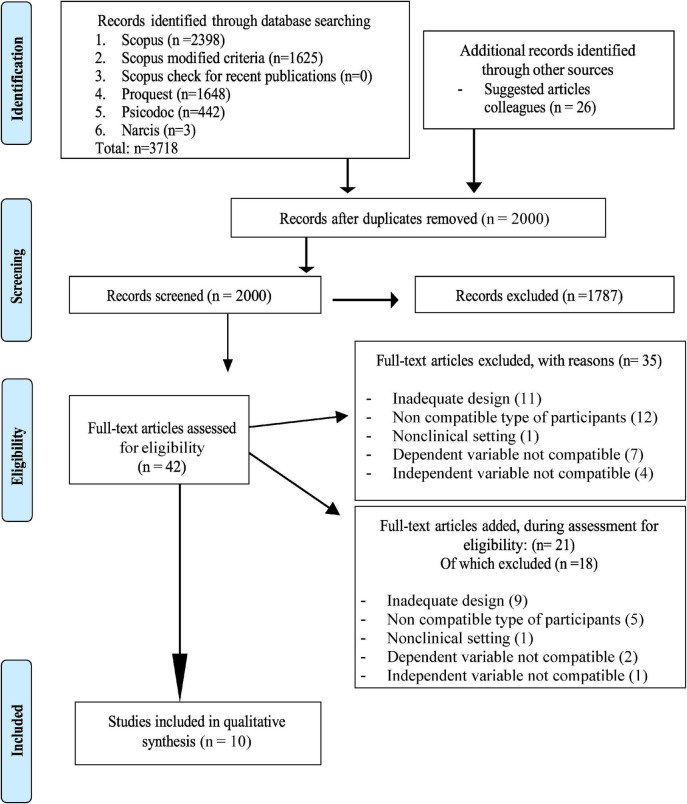
PRISMA 2009 flow diagram (24). Adapted with permission from the PRISMA team as stated on https://www.prisma-statement.org//PRISMA Statement/CitingAndUsingPRISMA.aspx.

In Figure 1, the PRISMA flowchart is presented to demonstrate the results during the screening process of the literature.

### Risk of bias

For the present review, one problem is evident. Participants across the studies differ to a large extent. Research on a clinical population, investigating humor interventions applied to participants, and measuring the effect on depression and/or anxiety was scarce. So other studies with a different clinical population, but investigating depression and anxiety were included. Even though we included only two studies that reported solely about schizophrenic participants, in the selection procedure this population appeared most frequently as a type of participant, which made us suspect that this population is more commonly used for investigating different types of interventions compared to participants with other mental health issues. That could contribute to the selection bias as other populations are less frequently used in clinical research.

The number of participants in the selected outcome studies varied between 20 and 40 participants. Although this number was sufficient for statistical purposes, questions can be raised whether the results also have clinical validity. In one correlational study (25) the number of participants was 110. In the two systematic reviews that we included, a total of 86 studies without stating the total number of participants (21) and 814 participants over 10 studies (22) were used. It is to be assumed that these are sufficient numbers. We should note that in both these review articles, the participants were very heterogeneous (see Table 1). A possible bias inherent in our systematic review is that we only present the findings on depression and anxiety outcome measures, whereas seven of the 10 included studies report on other outcome measurements as well. This could lead to reporting bias.

**TABLE 1:** Selected articles for qualitative synthesis.

References	Date	Type of study	Participants		
			Number	Age	Symptoms
Van der Wal and Kok (21)	2019	Systematic Review	Not specified.	All age.	Mental health and/or physical health issues. Measurement on depression and anxiety.
Zhao et al. (22)	2019	Systematic Review	814	Adults and elderly.	Schizophrenia, depression or physical health issues. Measurement on depression and anxiety.
Rudnick et al. (27)	2014	RCT	32	Not specified.	Mental illness (not specified). Measurement on depression and anxiety.
Ventis et al. (28)	2001	RCT	40 (2 male)	Undergraduate students.	Anxiety.
Deutsch (29)	2002	Quasi experimental	40	Age 21–64.	Depressive and non-depressive participants.
Cai et al. (17)	2014	RCT	30 (16 male)	Not specified.	Schizophrenia. Measurement on depression and anxiety.
Gelkopf et al. (26)	1993	RCT	22	Age 30–57.	Schizophrenia. Measurement on depression and anxiety.
O’Brien (10)	2001	Quasi experimental	20 (12 male)	Age 18–25.	Participants with distress. Measurement on depression and anxiety related items.
Panichelli et al. (25)	2018	Correlational	110 (40 male)	Age 20–70.	Diagnoses ranging from major depressive disorder to phobias and psychotic disorder. Measurement on depression.
Falkenberg et al. (16)	2011	Quasi experimental	6	Not specified.	Depression.

### Analytical approach

The included studies differed too much to perform a meta-analysis of the results (see Table 1). Therefore, we only performed a systematic review on all the included studies. In Table 2 the humor interventions used in each study are presented.

**TABLE 2:** Interventions and control groups.

References	Intervention	Comparison
Van der Wal and Kok (21)	- Laughter therapy with humor. - Laughter therapy without humor. - Laughter therapy, unknown whether humor was used.	All forms of control or comparison groups were allowed.
Zhao et al. (22)	- Laughter therapy. - Humor therapy. - Clown intervention.	Control groups received no specific humor or laughter intervention. Control conditions were for example: usual day care, placebo intervention.
Rudnick et al. (27)	- Stand-up comedy training (experimental arm). - Watching and discussing comedy videos (active control arm).	- Treatment as usual without any humor-related intervention (passive control arm).
Ventis et al. (28)	1. Systematic desensitization group (*n* = 13); rating non-humorous hierarchy items for fear, standard desensitization procedure. 2. Humor desensitization group (*n* = 14); rating non-humorous hierarchy items for fear, eliciting humorous perspective, completing incomplete statements about spiders in a humorous way.	- Control group (*n* = 12); no treatment. Waiting list condition.
Deutsch (29)	3 types of audiovisual stimuli: 1. “Static”: 10-min static signal, complete with white noise. 2. A nature documentary: this film was to provide interesting material for the subjects without being specifically humorous. 3. A comedy clip: a 10-min portion of a Seinfeld episode. - Each segment was 10 min long, divided into 2, 5-min segments, and viewed in 10 s increments. - Segments were run on individual basis to prevent group effects. - All subjects watched the 3 films in the order: nature documentary, comedy, static video.	- The static video was the control condition for the content-based formats of the other two conditions.
Cai et al. (17)	- *n* = 15 - Humor skill development program based on the 8 steps program by McGhee (37). - 5 weeks of training with 2 sessions a week.	- *n* = 15 - Doing handwork
Gelkopf et al. (26)	- The experimental group watched films labeled as comedies, such as Laurel and Hardy, Charlie Chaplin, Tootsie, and Police Academy. - During 3 months, daily 2 films, 4 days of the week.	- The control group watched neutral films (action, romantic, drama) and also some comedies (only 15% of the films). - During 3 months, daily 2 films, 4 days of the week.
O’Brien (10)	- 10 therapists treated 2 participants: 1 participant in the experimental condition, 1 in the control condition. - Humor defined as a verbal behavior with the elements of SLAP. Therapists were to remember the elements of humor for use in session and increasing the frequency with which comments containing SLAP were made. - Brief counseling was offered, limited to four sessions.	- Restricting the use of humor, therapists were to limit comments containing SLAP. - Brief counseling was offered, limited to four sessions.
Panichelli et al. (25)	- No interventions were designed to be applied. Humorous interventions were scored in retrospect. - Spontaneous humor was allowed during the sessions, but aggressive humor was avoided to protect the therapeutic alliance. - Humorous interventions were used only if clinically appropriate. - Various humorous interventions were used: * Exaggeration of the client’s ideas and behavior. * Expressing non-verbalized or implicit client thoughts. * Asking about the client’s favorite joke. * Using jokes and metaphors. * Giving a humorous, provocative nickname. - Sessions have been conducted by 1 therapist, the author of the study.	- No control
Falkenberg et al. (16)	- Humor training program McGhee.	- No control.

### Outcome measures

Because of the differences between the studies selected, the individual outcomes of each study are presented.

## Results

### Designs, countries, and languages used in studies

Of the 10 studies included in the analysis, two of them are review articles following the PRISMA guidelines. Four studies reported on randomized controlled trials (RCT). Three had a quasi-experimental design. One study was correlational (see Table 1).

Some of the studies in our review were also included in the systematic reviews. In the review by Van der Wal and Kok (21), the study of Cai et al. (17) is part of the 86 studies included in their qualitative synthesis. In the review by Zhao et al. (22), both the study of Cai et al. (17) and the study by Gelkopf et al. (26) are included in the selection of 10 articles to be analyzed. We have to conclude that there is some overlap, a bigger percentage for the Zhao review (20%), and a small percentage (1.16%) in the Van der Wal and Kok review.

Even though we searched for studies in Spanish and Dutch as well, only studies in English were included in the final analyses. The countries in which the research of the studies took place differ considerably: Belgium (1), Canada (1), China (2), Germany (1), Israel (1), The Netherlands (1), and USA (3).

### Type of participants

The focus of this systematic review is on adults (age 18–65) in a clinical setting with a mild depression or mild anxiety disorder. In the 10 studies that we selected, we found a rather diverse group (see Table 1). First, in the systematic reviews that we included (21, 22) participants differed considerably in age (children, adults, and elderly age 70) and in symptoms (for example, depression, anxiety, schizophrenia, Parkinson’s, or breast cancer). In the RCT studies, we also saw a mix of different types of participants, who were all adults: not specified mental illness (27), undergraduate students with an anxiety disorder (28) or with mild symptoms in wellbeing (10), depression (29), or schizophrenia (17, 26). Falkenberg et al. (16) included participants with a major depression in their quasi-experimental study. In the correlational study by Panichelli et al. (25), the same trend is being observed with participants suffering one or more of 12 different diagnoses ranging from depression, and anxiety to brief psychotic disorder.

### Type of interventions and control conditions

Two of the 10 studies (10, 25) contained therapeutic interventions applied by a therapist, such as: giving a humorous provocative nickname, using jokes and metaphors, exaggerating client’s ideas and behavior, or employing elements of SLAP (Surprise, Light-heartedness, Absurdity, and Perspective development) (see Table 2).

The other eight studies contained interventions that were not applied by a therapist in a one-on-one or a group therapy session with clients. Examples of these kinds of interventions are laughter induced by dancing, clapping, and laughing exercises (21), Humor Skill training (16, 17, 22, 27), watching humorous movies or programs (21, 22, 26, 27, 29), and systematic desensitization where participants were taught to elicit a humorous perspective and complete incomplete statements about spiders in a humorous way (28).

The study of Deutsch (29) had an experimental design in which depressive and non-depressive participants had to react to humorous material by pressing a lever to see more humorous material. Although the design was not clinical, outcome was measured on the Beck Depression Inventory II and the amount of laughing.

In all studies a control group was used, except for Falkenberg et al. (16) and Panichelli et al. (25). The first because it was a quasi-experimental study, the latter because it was a correlational study. In the systematic reviews (21, 22) all types of control groups were allowed. In the other six studies, treatment as usual (10, 27), a waiting list (28), and a neutral condition (17, 26, 29) were chosen as control conditions.

## Analysis of outcomes

### Depression

In seven studies, depression was an outcome measure (see Table 3): The systematic review by Van der Wal and Kok (21) showed that 26 of the 31 studies reported on depression. Depression decreased significantly. There was a larger effect size when all included studies were selected, compared to when only RCT studies were selected. For humor-induced laughter, the average effect size was 41% lower than for non-humor-induced laughter (*d*_*ppc*2_ = 0.43 vs. *d*_*ppc*2_ = 0.73). Non-humorous therapies showed an effect size twice as large as humorous therapies. However, questions were raised either about the replicability of the results or the clinical relevance of the significant, but small, reduction of outcome on the depression scales used (Beck Depression Inventory and Geriatric Depression Scale). The meta-analysis showed results similar to those of the systematic review.

**TABLE 3:** Results on depression studies.

References	Results on depression
	Decrease of symptoms
Van der Wal and Kok (21)	- In 26 of 31 studies. - Average effect size for humorous therapies was 41% lower than for non-humorous therapies.
Zhao et al. (22)	- In 9 of 10 studies. - Laughter and humor interventions (*p* = 0.001). - Laughter interventions alone (*p* < 0.0001) (6 studies). - Humor interventions alone (*p* = 0.34) (3 studies).
Deutsch (29)	- Significant for the non-depressed group (*p* < 0.05) on the BDI-II. - Not significant for the depressed group (*p* > 0.05) on the BDI-II.
Cai et al. (17)	- Measured on BDI (Chinese version) (*p* < 0.005).
Gelkopf et al. (26)	- Measured on BPRS total score (*p* < 0.001). - Measured on sub-score BPRS: Anxiety-depression (*p* < 0.001).
Panichelli et al. (25)	- Decrease on the Hamilton Depression Scale (*p* < 0.001). - Negative correlation for the CGI-2 (*p* < 0.001).
Falkenberg et al. (16)	- No significant long-term improvement on BDI (*p* = 0.17). - Significant improvement on cheerfulness STCI-S (*p* = 0.03) and STCI-T (*p* = 0.05). - Significant decreases on seriousness STCI-S (*p* = 0.03) and STCI-T (*p* = 0.05). - Significant decrease on bad mood STCI-S (*p* = 0.03), not on STCI-T (*p* = 0.12). - Significant improvement on mood after 6 of the 7 meetings measured with the VAS (varying between *p* = 0.01 and *p* = 0.05). Only the last meeting the improvement was not significant (p = 0.10).

BDI, beck depression scale; BPRS, Brief Psychiatric Rating Scale; CGI-2, clinical global impressions scales for global improvement; STCI-S and T, State and Trait Cheerfulness Inventory; VAS, Visual Analog Scale, measuring expectations and effectiveness of the humor training on their mood as perceived by the participants.

In the Zhao et al. (22) systematic review nine of the 10 studies reported on depression. A significant effect of the interventions on depression was found in four of them. Laughter and humor interventions provided a statistical improvement in depression (*p* = 0.001) with a small effect size pooled across studies. Six studies provided data on laughter therapies. A statistical improvement was found (*p* < 0.0001), with a medium effect size pooled across studies. Three studies provided data on humor therapies. No significant intervention effect was found (*p* = 0.34).

Deutsch (29) reports in his experimental study that a small but significant correlation was found between pre-test and post-test scores on the Beck Depression Inventory-II for the non-depressed group: *t*(19) = 2.163 (*p* < 0.05) indicating a decrease of depressive symptoms. For the depressed group, no significant differences were found: *t*(19) = –0.38 (*p* > 0.05). It was not specified which of the three conditions (humorous, non-humorous, or control condition audio-visual material) contributed to what extent to these results.

In the study by Cai et al. (17), a decrease was found in the depression score [*F*(1, 28) = 18.89; *p* < 0.005] in the humor group. Participants in the control group reported no changes in the depression score.

Gelkopf et al. (26) report that significant results were found for the total Brief Psychiatric Rating Scale score (*p* < 0.001) as for anxiety-depression (*p* < 0.001), which indicates that in the humor experimental group there was a decrease in the total number of observed psychiatric symptoms, especially those concerning anxiety and depression. These scores were based on the judgment of a psychiatrist. Self-scores by participants on anxiety or depression were significant.

Panichelli et al. (25) found that scores on the Hamilton Depression Scale significantly decreased (*p* < 0.001) from 10.8 ± 5.8 (range 1–29) at session 1 to 7.9 ± 5.2 (range 0–23) at session 10 or later, which accounted for a Hamilton difference score of 2.9 ± 3.1 (range 7–12). Furthermore, a negative correlation was found with the Clinical General Impressions scale-2 score: r_*s*_ = –0.37 (*p* < 0.001), indicating higher ratings of the presence of humor in the therapy when participants improved more according to the clinical impression of the therapist. No correlation was found with Hamilton difference scores. But when applying a multiple regression analysis between session 10 Hamilton scores and client ratings of the presence of humor during sessions, a significant negative association between both parameters was found when adjusted for session 1 Hamilton scores (*p* < 0.001). This same pattern was observed as seen from the therapist’s perspective (*p* < 0.01). In the subgroup of clients who reported a high frequency of therapist-initiated humor (score > 2; 4 maximum; *N* = 45) clients who rated the therapist’s humor as less funny (score 0–2; *N* = 11) were compared with those who rated it as funnier (score 3–4; *N* = 34).

Falkenberg et al.’s (16) study results showed that there was no significant long-term improvement of depressive symptoms: Z = –1.4, *p* = 0.17 on the Beck Depression Inventory. Although the State and Trait Cheerfulness Inventory and the Visual Analog Scale were not designed to measure depression itself, cheerfulness and mood are frequently related to depression (30, 31). The improvement in both state (Z = –2.2, *p* = 0.03) and trait cheerfulness (Z = –1.9, *p* = 0.05) proved to be significant. Meanwhile, decreases were significant in both state (Z = –2.2, *p* = 0.03) and trait seriousness (Z = –1.9, *p* = 0.05) and state (Z = –2.2, *p* = 0.03) but not trait (Z = –1.57, *p* = 0.12) bad mood. In the sessions, short-term mood improvement was achieved.

### Anxiety

Five studies reported results on anxiety (see Table 4). In Van der Wal and Kok (21), anxiety was measured in 15 studies. In 14 of them, anxiety decreased significantly after the laughter-inducing intervention. The average effect size for humorous therapies was 49% lower than for non-humorous therapies (*d*_*ppc*2_ = 0.51 vs. *d*_*ppc*2_ = 1.00).

**TABLE 4:** Results on anxiety studies.

References	Results on anxiety
	Decrease of symptoms
Van der Wal and Kok (21)	- In 14 of 15 studies. - Average effect size for humorous therapies was 49% lower than for non-humorous therapies.
Zhao et al. (22)	- In 2 of 7 studies. - Laughter and humor interventions (*p* = 0.01). - Laughter interventions alone (*p* = 0.02) (5 studies). - Humor interventions alone (*p* = 0.28) (2 studies).
Ventis et al. (28)	- Significant for humorous and non-humorous interventions compared to control group: - Finished more items on BAT (*p* = 0.006). No significant difference between both type of interventions. - Scored higher on SCD (*p* = 0.001). No significant difference between both type of interventions. - On fear ratings, only non-humorous interventions had significant results (*p* = 0.49).
Cai et al. (17)	- Measured on State Trait Anxiety Inventory (Chinese version) (*p* < 0.005).
Gelkopf et al. (26)	- Measured on BPRS total score (*p* < 0.001). - Measured on sub-score BPRS: Anxiety-depression (*p* < 0.001)

BAT, Behavioral Approach Test; SCD, Spider Cognitive-Dimensions; BPRS, Brief Psychiatric Rating Scale.

Zhao et al. (22) reported on seven studies about the anxiety of which two found a significant effect. A significant improvement in anxiety was found (*p* = 0.01) with a medium effect size of laughter and humor interventions. Five studies reported on laughter interventions. A significant result was found (*p* = 0.02). In the two studies that reported on humor interventions no significant result was found (*p* = 0.28).

The Ventis et al. (28) study shows that the two treatment groups (humorous and non-humorous systematic desensitization) showed significantly greater post-test scores on three measures: First of all, both groups completed significantly more items on the Behavioral Approach Test than the control group did [*F*(2, 36) = 5,95, *p* = 0.006]. But the groups did not differ from each other. Then, both groups also exhibited significantly higher post-test scores on their Spider Cognitive-Dimension ratings, than the control group did [*F*(2, 35) = 8.00, *p* = 0.001]. And again, no differences were found between the two groups. Only the systematic desensitization group differed significantly on the fear ratings [*F*(2, 36) = 3.29, *p* = 0.049]. The humor desensitization group showed numerically similar results, but apparently not significant.

Cai et al. (17) report a decrease in the anxiety [*F*(1, 28) = 27.11; *p* < 0.005] score in the humor group. In the control group, participants did not report changes in the anxiety score.

The results of Gelkopf et al. (26) on anxiety were described in the depression section above.

### Not specified

In two studies (see Table 5), it was not clear whether depression or anxiety was measured, because of the use of transdiagnostic diagnosis in which there was no differentiation between disorders (27) or because the researcher used instruments measuring generalized discomfort or distress and the degree in which the problems bothered the participants rather than depression or anxiety explicitly (10).

**TABLE 5:** Results on unspecified depression or anxiety studies.

References	Results on studies not specified on depression/Anxiety
	Decrease of symptoms
Rudnick et al. (27)	No significant results were found for attrition of mental health measures.
O’Brien (10)	- A decrease of symptoms of distress measured on the Hopkins Symptom Checklist-58 for both humorous and non-humorous conditions was significant (*p* < 0.06). - A decrease of problem distress based on global judgments of therapist and participant was only significant for the humor condition (*p* < 0.06).

Rudnick (27) reported that no significant results were found for attrition of mental health measures. There was only a marginally significant interaction effect for one of the experimental conditions with humor and time for self-esteem scores. The principal author of the study confirmed, after consulting him, that they did not explain what type of mental health measures were involved because it concerned a transdiagnostic research.

In the study by O’ Brien (10), both participants and therapist could distinguish between sessions in which more or less humor was used. But there the study failed to show a significant difference between the two conditions. A decrease of symptoms of distress measured on the Hopkins Symptom Checklist-58 for both conditions was significant, *t*(9) = 5.64, *p* < 0.06 (humor) and *t*(9) = 3.21, *p* < 0.06 (non-humor). A decrease of problem distress based on global judgments by both the therapist and the participant measured on a 9-point Likert scale was only significant for the humor condition, *t*(9) = 3.61, *p* < 0.06.

## Discussion

Several authors consider humor as a useful tool in psychotherapy (1, 7, 14, 19, 25, 32). We intended to present an overview of empirical evidence-based studies on all types of humor interventions and not just humor-related constructs like banter (6) and laughter (21, 22). Our second goal was to present the available empirical data showing the effect of humor interventions on levels of depression and anxiety. Furthermore, we wanted to assess the empiric support of humor as a clinical intervention in psychotherapy according to the SIGN system (20). Based on the systematic reviews, the positive effects of humor on a decrease of depression seem present but less than the positive effects of the control groups which consisted of interventions that were not humorous (21), or no significant effect could be established for humorous interventions (22). Regarding the RCT, we can observe that three of them show a significant effect for humor interventions (16, 17, 25, 26, 29). However, in the Falkenberg (16) study, the main depression scale failed to show a significant effect. And although a significant effect was found in the study by Deutsch (29), it was only found in the non-depressive group of participants.

Regarding the effect of humor on anxiety, the same trend was noticeable in the Van der Wal and Kok (21) study. Humorous interventions showed a significant effect size, but the non-humorous interventions had an effect size twice as large. In the Zhao et al. (22) study, the laughter and humorous interventions together had a significant effect size. But when divided into a subgroup of laughter interventions and a subgroup of humorous interventions, only the laughter interventions continued to show this significant effect size. Of the four RCT studies on anxiety (17, 26–28), only the Rudnick (27) study failed to show a significant effect on a decrease of anxiety. In the Ventis (28) study, three scales measured the effect on anxiety. On two scales, a significant result was found for both the humorous and neutral treatment groups. On the third, only the neutral treatment group was significant.

We have found that differently designed humorous interventions in different populations tend to show significant effects on a decrease of both depression and anxiety in several studies. At the same time, the variety in the design of the studies mirrors the image pictured in the introduction of this review that there is a great variety in viewpoints on the use of humor in psychotherapy. This is also being reflected in the selection procedure for this systematic review. First of all, we broadened the scope of participants in our systematic review. Second, both studies that included humorous interventions applied by a therapist only and studies in which participants were taught to apply humor themselves were included. Using the SIGN system (20) to assess the empirical support of the studies incorporated in our systematic review, we came to the conclusion that a B would be appropriate. It is important to note that this is due to the individual assessments of the studies. As a whole, there is still a high level of inconsistency between the studies regarding design, participants, and operationalization [*We use the term “operationalization” to refer to the process of turning abstract, in our case “humor,” concepts into measurable observations*]. Therefore, conclusions based on this review are not directly applicable in the clinical field but should be regarded as noteworthy information to continue the research in this area.

With such low consistency in the design of the studies, what are the challenges in the field of investigating humor according to our findings? First of all, the operationalization of humor. The definition of humor has an important impact on the operationalization in empirical research. In the selected studies we have observed humor with or without laughter, clown intervention, stand-up comedy training, watching comedy movies with or without discussing them, desensitization training applying humorous outlooks on the object of fear, humor skill training and humor applied by therapists containing elements of provocative therapy, or for example making comments which contained elements summarized by the acronym SLAP: surprise, lightheartedness, absurdity, and perspective development (10). Some of these interventions require the clients to produce humor themselves, others put them in a position of consuming or undergoing humor. A fundamental question that arises is, do clients need to produce humor themselves or not for it to be effective? According to Mindess (33), a shift from passive to active absorption of humor is very important. In a passive way, people may be able to reproduce humor, in an active way people might start to see things clearly and apply a humorous outlook on life themselves. This can be linked to the psychological flexibility in the ACT. Clients can sometimes be unable to distance themselves from their own situation to put into a broader context what happened to them and what they can do or change (7). Increasing psychological flexibility would mean that clients would be better able to distance themselves just like when producing humor. Another question is whether clients need to laugh as a result of the humor, i.e., do they need to find the humor funny to be effective? This question can be illustrated by the findings in Gelkopf et al. (26) and Panichelli et al. (25) where positive effects of the use of humor were found even when participants rated the particular sessions as less funny. Furthermore, how effective is humor if it is not directed specifically at the personal problems clients are dealing with, as is the case in the interventions where clients were presented with comedy movies or funny videoclips? Because of the differences in operationalization in the selected studies, it is not possible to say if they measured the same construct. Therefore, the moderate positive findings in this systematic review should be regarded with even more prudence.

Moreover, the design of the studies can have an important impact on the results found. Not only did we find differences in operationalizations of humor, but the participants differed as well. Although we selected studies in which a clinical setting was applied, participants differed in symptomology (ranging from distress to depression, anxiety, or even schizophrenia) and in the way they were recruited (ranging from students in undergraduate courses to chronic patients in a hospital ward). The number of participants was low in three studies (6, 20, and 22 participants). In the others, the number of participants ranged between 30 and 110. Only in the systematic reviews that we used data of larger numbers of participants were incorporated. Therefore, it is difficult to draw clear conclusions based on these findings.

Another point of criticism on the selected studies is that one had an experimental design, two a quasi-experimental design, one had a correlational design, five were RCT and two were systematic reviews. The inferences one can make based on these different designs are very different. Even though the results of these differently designed studies are presented together, we do not want to pretend that their conclusions can be added up.

And finally, in the study of Panichelli et al. (25), data were analyzed in retrospect, making reporting bias and confounding factors possible which could not be controlled and presenting a possible risk for the validity of the findings.

## Conclusion

The empirical data presented in this review of the present literature about humor in psychotherapy offers interesting perspectives about its potential psychotherapeutic effects on depression and anxiety. However, more research taking into account the above suggestions for improving the research design is needed. It would be valuable to investigate the effects of stimulating participants to actively produce humor compared to the situation in which participants are passive recipients of humor. Also of interest is the question of whether a difference might be observed when humor interventions of the therapist are directed at the client’s presenting problem, or whether the therapist’s humor is directed at anything else. The first might be a more risky move for the therapist because of the strain it can put on the therapeutic relationship, but could have more impact and therefore be more effective for the client. The latter could be “safer” for the therapist and the client but could be beneficial in lowering anxiety and establishing and enhancing the therapeutic alliance. An important starting point should be a clear operationalization, i.e., using a consistent way of measuring humor in psychotherapy. Although Martin (1) suggested that the reversal theory of humor could be seen as a future framework for an integrative theory of humor, in our opinion it is too early to integrate different viewpoints while within the individual theories there still is a lack of well-designed RCT research. At some point, an integration approach could prove to be the optimum solution, because probably the best way to view the concept of humor is the same as approaching the multi-facetted construct of IQ. Different aspects of humor can all contribute in some way to the wellbeing of clients with mental health problems. Thus, interventions based on a cognitive approach of humor will focus on other dimensions of humor and its application in psychotherapy than an emotional or a behavioral approach. Similarly, interventions designed to stimulate clients to produce humor themselves will have other effects on them than interventions in which they are more passive recipients of humor. They all can be beneficial, and the different approaches might be combined. But let us first investigate the different aspects before integrating them.

To continue this investigation to humor and to integrate its different aspects we propose an approach to humorous interventions in psychotherapy based on Koestler’s (34), Sul’s (35), and Shultz’s (36) approaches. A humorous intervention in psychotherapy contains surprise which will make clients confused for a moment. Because of this confusion clients are invited to doubt their habitual form of approaching their life and must search for an alternative framework to resolve the confusion. In this process, clients are taking on new perspectives, have a chance to distance themselves from the actual situation, and feel relieved when they discover a solution or realizes that what first seemed to be a problem, stopped being one. Most commonly clients recognize the intervention as a humorous one and/or have to laugh. However, an intervention can be humorous without clients recognizing it and having to laugh because of it. This framework is intended to initiate wider research to the application of humor in psychotherapy. There is, of course, a possibility that based on the results of future research, this definition needs to be modified.

## Data availability statement

The original contributions presented in this study are included in the article/supplementary material, further inquiries can be directed to the corresponding author.

## Author contributions

FS and JG-M conceived the study. FS wrote the initial draft and all tabular material. JG-M supervised the study and critically revised the manuscript. Both authors read and agreed to the published version of the manuscript.

## Footnotes

1 This review is part of a Ph.D thesis on the role of humor on the psychological flexibility of patients with a mild depression or anxiety disorder. Our goal therefore was to present a systematic review on humor interventions and the effect on psychological flexibility, depression, and anxiety.
